# Molecular Genomics of Oral Submucous Fibrosis: A Narrative Review

**DOI:** 10.3390/genes16060612

**Published:** 2025-05-22

**Authors:** Vasileios Zisis, Stefanos Zisis, Christina Charisi, Konstantinos Poulopoulos, Aristeidis Sarkisian, Athanasios Poulopoulos

**Affiliations:** 1 Department of Oral Medicine/Pathology, School of Dentistry, Aristotle University of Thessaloniki, 54124 Thessaloniki, Greeceakpoul@dent.auth.gr (A.P.); 2 Clinic for Operative Dentistry, Periodontology and Preventive Dentistry, University Hospital RWTH Aachen, 52074 Aachen, Germany

**Keywords:** oral submucous fibrosis, oral cancer, oral potentially malignant disorder, oral precancer, oral lesion, genomics, proteomics

## Abstract

Background: Oral Submucous Fibrosis (OSMF) is a chronic, progressive condition characterized by the fibrosis of the oral mucosa, often associated with the habitual consumption of areca nut and tobacco, leading to significant morbidity. Despite its prevalent occurrence in many parts of the world, the underlying genetic and molecular mechanisms remain poorly understood, highlighting a critical need for research into its molecular genomics. The aim of this literature review is to investigate the molecular genomics of Oral Submucous Fibrosis by analyzing the relevant literature of the past decade. Methods: The search was conducted using MEDLINE (National Library of Medicine)-PubMed, focusing on the period 2015–2025 using the following keywords: Molecular Genomics AND Oral Submucous Fibrosis. This was followed by a manual search, and references were used to identify relevant articles. Results: A total of 12 articles were included in our review according to our inclusion criteria, which illustrated the importance of TGF-β, Wnt inhibitory factor-1, CypA, Hsp-70 1B, Calreticulin, Lumican, Enolase 1, MMP-2, IGF-1R, XIST, Epigallocatechin-3-gallate, Von Hippel-Lindau, and MUC1 and 4. Conclusions: Understanding the molecular pathogenesis of OSMF involves examining the molecular interactions and the roles of specific proteins. Advanced genomic technologies have opened new frontiers in the study of OSMF. As research in OSMF continues to evolve, emerging interdisciplinary approaches may provide therapeutic strategies, aiming to improve management outcomes for the patients.

## 1. Introduction

The prevalence of Oral Submucous Fibrosis (OSF) is shaped by regional patterns of substance use, largely due to cultural practices surrounding the consumption of betel quid and areca nut, and demographic characteristics [1]. According to the World Health Organization, approximately five million people worldwide are afflicted by OSF [1]. In India, the prevalence of OSF has surged from 0.03% to 6.4% over the last four decades [1]. The northern part of the country exhibits the highest rates, ranging from 30% to 42%, while the western part of the country reports much lower rates, from 0.03% to 0.2% [1]. There is a slight female predilection, with rates for males between 0.2 and 2.3% and females between 1.2 and 4.57% [2]. OSF is observed in Asian populations, including Asian immigrants in the UK and South and East Africa [2,3]. At least 95% of individuals who develop OSF are smokers [4]. Also, smokers who consume a higher number of cigarettes daily are at a heightened risk of developing airflow obstruction [4]. The motivation to quit smoking is higher when smokers recognize the danger of developing smoking-related diseases [4]. The frequency and duration of betel quid chewing elevate the risk of developing OSF [5,6]. The psychoactive properties of areca nut encourage its habitual use and contribute to the frequent consumption [6]. Areca nut may also be integrated with tobacco products, with combined effects [6]. One initial symptom is the gradual limitation of mouth opening, known as trismus (exacerbated by the formation of taut bands) [7,8]. This inability to open the mouth affects both speech and eating habits [7]. Patients also report burning mouth syndrome, and depending on the dietary habits, it may be aggravated by the consumption of spicy foods [7,8]. The stiffening of the oral mucosa and oropharynx limits mouth movements [7,9]. Blanching of the oral mucosa is observed, due to fibrotic changes [7,10] (upper and lower labial mucosa, soft palate and faucial pillars, display discoloration, accompanied by the development of palpable, thick fibrous bands, extending to attached gingiva and floor of the mouth, leading to a loss of surface texture and elasticity [11]). Ulcerations and vesiculation may also emerge [7]. The fibrotic changes lead to vertical bands in the soft tissues, which restrict the ability to open the mouth [11]. A brownish-black pigmentation in the posterior vestibular region may indicate the chronic nature of the disease [11]. Xerostomia (dry mouth) has also been reported [12]. The restricted movement is often compounded by the presence of fibrous bands in the cheeks and lips, contributing to the stiffness and limited functionality of the oral cavity [12]. These physical limitations can lead to problems with speech and swallowing, further affecting the patient’s quality of life [12]. Swallowing difficulties may also be exacerbated by other symptoms such as a burning sensation when consuming spicy food and a dry mouth, which are commonly reported by patients with OSMF [12].

The restricted mouth opening may blur the diagnostic process. Scleroderma provokes a similar clinical appearance; however, the burning sensation, ulceration, and pain of the oral mucosa are not typically seen in scleroderma [13]. Scleroderma presents with the typical purse-string-like appearance around the mouth and microstomia due to collagen deposition in the perioral region [13]. The systemic scleroderma affects skin, blood vessels, and visceral organs, in contrast to the exclusively intraoral localization of OSF [13]. Even if patients cease to consume betel quid, it does not guarantee complete recovery [1].

The aim of our review is to summarize the findings of the original research articles of the past decade, thus providing the readers with a thorough update of Oral Submucous Fibrosis.

## 2. Materials and Methods

An electronic search of literature was performed in March 2025 to identify all articles investigating the Molecular Genomics of Oral Submucous Fibrosis. The search was conducted using MEDLINE (National Library of Medicine)-PubMed with restrictions concerning the date of publication. In particular, we focused on the period 2015–2025 using the following keywords: Molecular genomics AND Oral submucous fibrosis. This was followed by a manual search, and references were used to identify relevant articles. The articles identified from the electronic and manual search were screened to eliminate those that failed to meet the respective inclusion and exclusion criteria as listed below.

### Inclusion and Exclusion Criteria

The electronic search is based on the following inclusion criteria:

1. Language: Articles written in English;
2. Time frame: Studies published between 2015–2025;
3. Type of study: Original research articles.

The following exclusion criteria were applied:

1. Language: Articles written in a language other than English;
2. Time frame: Articles written before 2015;
3. Type of study: Non-original research articles.

## 3. Results

**Figure 1:**
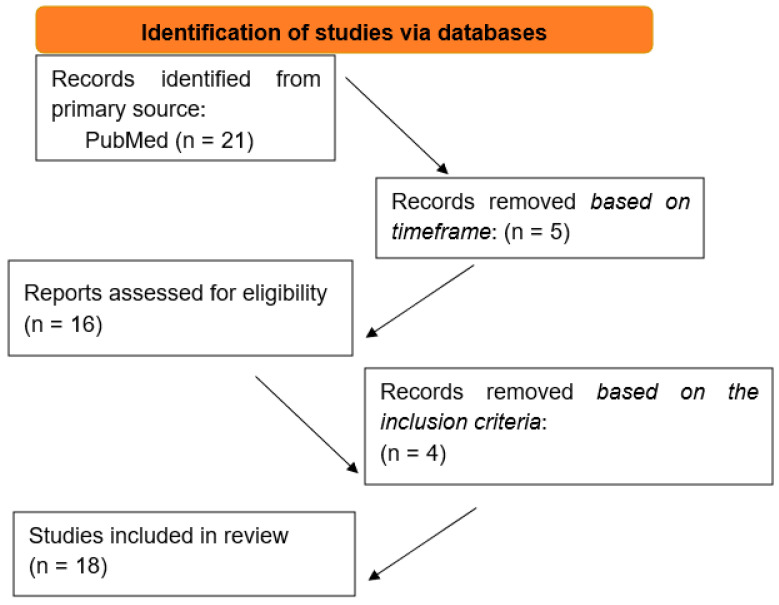
Flowchart of our study.

In total, 21 articles were identified through the keywords. After the implementation of the time frame 2015–2025, 16 articles remained. Subsequently, by reading the titles and abstracts, and therefore by excluding the non-original research articles and the articles written in a language other than English, 12 articles remained (Table 1, Figure 1).

The features of the included articles are summarized in Table 2.

Areca nut induces the secretion of certain factors which lead to a myofibroblast phenotype similar to OSF. TGF-β is the causative agent of this phenotype in terms of the areca nut-induced secretome [14]. Promoter methylation often causes the downregulation or even silencing of the Wnt inhibitory factor-1 in the carcinogenesis of OSF, which in turn promotes the early detection of OSCC by serving as a potential epigenetic biomarker [15]. OSF induces an up-regulation of CypA, and the expression level of the latter is correlated with the progression of the former and, more specifically, its malignant transformation. Fibroblast cell proliferation is regulated by modulating CypA expression, and conversely, apoptosis is caused in human fibroblasts when CypA is lost. Furthermore, ATK, ERK and Caspase 3 are regulated by CypA, and the mitochondrial membrane potential is reduced by it (a general disorder of mitochondria is being shown when apoptosis is induced as their higher permeability leads to the loss of the regulatory state of oxidative phosphorylation, leading to the release of factors with pro-apoptotic effects and thus mitochondrial membrane potential negation) [16]. Another study supports the same finding, that CypA is the OSF-associated protein candidate [17]. Hsp-70 1B, Calreticulin, and Lumican variant were significantly increased (6.2-, 3.3-, and 2.8-fold, respectively), whereas Enolase 1 was decreased by 0.5 fold in the OSMF tissues [18]. The invasion and migration of OSCC is induced by OSF, which, furthermore, activates MMP-2 in OSCC. However, cell proliferation and tumor growth of OSCC are reduced in terms of OSF, meaning that perhaps in such cases, OSF-induced OSCC has a better prognosis than the regular OSCC. Finally, OSF activates the epithelial to mesenchymal transition of OSCC, thus promoting the EMT-induced invasion by upregulating IGF-1R expression in human oral cancer (IGF-1R is increased in OSCC patients) [19]. The exome sequencing constitutes an interesting preliminary method of navigating the genetic landscape of rare care reports [20]. XIST is a non-coding RNA transcribed from the X chromosome and acts as a major effector of the X-inactivation process. The hypermethylation of the XIST promoter downregulates the transcript for the XIST gene [21]. Thereby, the X chromosome genes are dysregulated in OSF tissues. In total, 38 downregulated-hypermethylated genes and 55 hypomethylated-upregulated genes were observed. Increased expression of FGF13, RPS6KA3, and ACSL4. Insulin signaling, ubiquitin-mediated proteolysis, nicotine addiction, and RAS/MAPK pathways were dysregulated [21]. Epigallocatechin-3-gallate inhibits the process of oral carcinogenesis [22]. A recent study determined a statistically significant association of GSTT1 gene polymorphism with OSF [23]. Von Hippel-Lindau (VHL) was downregulated due to hypermethylation in OSF, whereas the overexpression of VHL inhibited the differentiation of oral fibroblasts into myofibroblasts. The promoter of VHL was methylated in OSF. DNA methyltransferase 3A induced the hypermethylation of the VHL promoter. VHL could promote the ubiquitination of tenascin-C. Overexpression of tenascin-C notably reversed the inhibitory effect of oe-VHL on the differentiation of oral fibroblasts into myofibroblasts [24]. MUC1 and 4 allow for the monitoring of both OSCC and OPMD. MUC1 is highly expressed in undifferentiated grades, indicating a poorer prognosis, and MUC4 is highly expressed in more differentiated tumors, indicating a better prognosis. These markers, combined, help distinguish lesions in both extremes [25].

Based on the analysis of the role of the markers (Table 3), the most important markers may be considered to be TGF-β, due to the associated signaling, CypA, MUC1, and MUC4, because their presence determines the prognosis of both premalignant and malignant lesions.

## 4. Discussion

The identification of key genetic markers associated with OSF is crucial for understanding its pathogenesis and potential for malignant transformation. Sister Chromatid Exchanges (SCEs) constitute primary genetic markers, since they indicate the potentially malignant genetic instability of OSF [26]. HLA genotypes play a crucial role as well, suggesting a genetic predisposition to develop OSF [26]. Moreover, the Transforming Growth Factor Beta (TGF-β) induces the characteristic fibrotic reactions of OSMF [6]. TGF-β is significantly stained in early OSF cases and contributes to the epithelial–mesenchymal transition (EMT) that leads to fibrosis [6]. The uncontrolled TGF signaling is a critical factor in the fibrotic process [6]. The pathogenesis of OSF is linked to EMT. EMT plays a crucial role in the development of extracellular matrix (ECM)-producing cells, such as fibroblasts and myofibroblasts, which in turn lead to fibrosis characteristic of OSF [27]. Key genes involved in this transition, including SFRP4, THBS1, MMP-2, and CLC-18, are induced by TGF-β, highlighting potential intercorrelations among EMT, TGF, and OSF [27]. Additionally, transcription factors like Snail1 and Snail2 (Slug) regulate genes linked to EMT, further exacerbating the fibrotic process associated with OSF [27]. Arecoline, a major component of areca nut, increases the expression of Twist in buccal mucosal fibroblasts, promoting a myofibroblastic phenotype through EMT and thereby accelerating the fibrotic changes seen in OSF [27]. The EMT process in OSF is characterized by alterations in cellular markers, such as reduced levels of E-cadherin and β-catenin and increased levels of N-cadherin and Twist, which are associated with the progression of the disease [27]. The development of Oral Submucous Fibrosis (OSMF) is intricately linked to alterations in molecular pathways that influence cellular and extracellular dynamics. The central key to the development of OSF is the transformation of myofibroblasts into their altered counterparts with a protumorigenic phenotype [28]. These altered myofibroblasts play a pivotal role in reorganizing the extracellular matrix (ECM) and producing inflammatory mediators, which lead to fibrosis [28]. The dysregulation of collagen metabolism is a hallmark of OSF, mediated by enzymes such as collagenase and lysyl oxidase (collagen dysregulation means problematic ECM remodeling) [29]. EMT thrives in this hypoxic, fibrotic microenvironment and allows for the acquisition of a mesenchymal phenotype by epithelial cells, predisposing them to epithelial malignancy [28]. Inflammatory cells and cytokines influence the molecular interactions within the fibrotic milieu of OSF [30]. Osteopontin (OPN) upregulates the inflammatory response and is involved in the fibrosis and potential malignancy of OSF [30]. OPN’s influence extends to the fibroblast–myofibroblast transition, a critical step in fibrosis, where it enhances the activation of Transforming Growth Factor-Beta (TGF-β) by stimulating mast cell granulation [30]. Here, we may witness even more intercorrelations among EMT, TGF, OPN, and OSF. OPN initiates and promotes the fibrotic stages [30]. The interplay between OPN and other profibrotic mediators influences the structural changes in OSF [30]. Wnt signaling pathway proteins, particularly SFRP1 and SFRP5, are crucial as their reduced levels due to promoter methylation lead to cytoplasmic and nuclear accumulation of β-catenin, facilitating tumor progression [6]; thereby, these proteins may serve as biomarkers for malignant progression [6]. PTEN, a tumor suppressor gene, is implicated in OSF and characterized by the progressive loss of expression from normal oral mucosa to OSF and subsequently to oral squamous cell carcinoma (OSCC) [31]. This loss of expression is not statistically significant between early and advanced OSF; however, it suggests a mechanism that mediates the malignant transformation of OSF [31].

Potential therapeutic targets include TGFβ, TGFα, TNFα, HIF-1α, and fibroblast growth factor (FGF), indicating the diverse molecular pathways involved in OSF pathogenesis [32]. Matrix metalloproteinases (MMPs) and tissue inhibitors of metalloproteinases (TIMPs) may also serve as potential therapeutic targets due to their involvement in extracellular matrix remodeling [32]. Additionally, SMAD proteins are involved in signal transduction pathways that mediate fibrotic responses [32]. The modulation of extracellular matrix deposition through local injections of enzymes such as hyaluronidase, collagenase, and chymotrypsin may reduce ECM deposition [33]. The use of immunomodulators, including steroids and anti-fibrotic cytokines, can effectively inhibit inflammation and prevent the activation of myofibroblasts [33]. Polyphenols, particularly epigallocatechin-3-gallate (EGCG), modulate protein targets such as Transforming Growth Factor Beta-1 (TGF-β1) and lysyl oxidase (LOX), which constitute vital components in the fibrotic pathway [34]. The polyphenols may both downregulate TGF-β1 and collagen mRNA expression [34]. Pentoxyfilline exhibits vasodilating properties and reduces inflammatory mediators [35]. Pentoxyfilline administration over a three-month period results in statistically significant improvements in OSF-related symptoms, including mouth opening and burning mouth syndrome [35].

Organoid technology enhances the study of OSF by providing a model to achieve in vitro in vivo extrapolation [36]. The application of human umbilical vein endothelial cells (HUVECs), fibroblasts, and epithelial organoids allows for the recreation of the interaction between the epithelium and mesenchyme [36]. This recreation of OSF revealed the regulatory role of thrombospondin-1 (THBS1) and, in particular, that stromal thrombospondin 1 suppresses angiogenesis in Oral Submucous Fibrosis [36].

The multifaceted nature of OSF necessitates collaboration among geneticists, oral pathologists, and clinical practitioners to allow for better management of the disease [37]. The cessation of betel quid, which is a main etiological factor of OSF, may be more effectively managed through the combined efforts of dental professionals, psychologists, and addiction specialists [37]. Early diagnosis and intervention remain critical because they ameliorate symptoms and mitigate the risk of progression to oral cancer [11].

## 5. Conclusions

Understanding the molecular pathogenesis of OSMF involves examining the molecular interactions and the roles of specific proteins. Advanced genomic technologies have opened new frontiers in the study of OSMF. As research in OSMF continues to evolve, emerging interdisciplinary approaches may provide therapeutic strategies, aiming to improve management outcomes for the patients. Our review yielded several markers, the most important of which were, according to our understanding, TGF-β, due to the associated signaling, CypA, MUC1, and MUC4, because their presence determines the prognosis of both premalignant and malignant lesions. These markers could be implemented in clinical practice in an effort to distinguish the most prognostically dangerous lesions from the rest.

## Figures and Tables

**Table 1:** Studies included in the comprehensive review.

21 Articles Through Keywords	
Time frame 2015–2025	
16 articles remained	5 articles excluded
The rest of the inclusion criteria	
12 articles remained	4 articles excluded

**Table 2:** Summary of the main findings and characteristics of the original studies included in our comprehensive review.

Study	Country	Year	Sample Size	Method
[14]	India	2015	-	RT-PCR, microarray, data analysis, and immunohistochemistry
[15]	China	2015	45 (OSF)	RT-PCR, methylation-specific PCR, and immunohistochemistry
[16]	China	2016	60 (OSF)	Two-dimensional electrophoresis-based proteomic approach, western blot, and immunohistochemistry
[17]	China	2017	-	Two-dimensional electrophoresis-based proteomic approach, western blot, and immunohistochemistry
[18]	India	2018	63 (OSF)	Two-dimensional electrophoresis-based proteomic approach, immunohistochemistry, and RT-PCR
[19]	Taiwan	2021	3 (OSF)	IHC, RT-PCR, Cell assay, and western blot
[20]	India	2022	1 (OSF)	Exome sequencing of a rare case report
[21]	India	2022	7 (OSF)	MS- PCR and RT- PCR
[22]	China	2023	3 (OSF)	Functional annotation and pathway enrichment analysis.
[23]	Pakistan	2024	108 (OSF)	DNA extraction and PCR amplification.
[24]	China	2024	32 (OSF)	Cell culture, MS-PCR, RT-PCR, western blot, and immunohistochemistry.
[25]	India	2025	63 (OSCC, OPMD, OSF, NOM)	RT-PCR and immunohistochemistry.

**Table 3:** The role of each marker, associated with OSF.

Markers	Role
TGF-β	Signaling pathways
WNt IF-1	Signaling pathways
MMP-2	Signaling pathways
IGF-IR	Signaling pathways
Insulin signaling	Signaling pathways
Ubiquitin proteolysis	Signaling pathways
RAS/MAPK	Signaling pathways
EGCG	Signaling pathways
GSTM1	Gene polymorphism
GSTT1	Gene polymorphism
VHL	Epigenetics
FGF13	Epigenetics
RPS6KA3	Epigenetics
ACSL4	Epigenetics
XIST	Epigenetics
CypA	Proteomics
Hsp-70	Proteomics
Calreticulin	Proteomics
Lumican	Proteomics
Enolase 1	Proteomics
MUC1	Proteomics
MUC4	Proteomics

## Data Availability

The original data presented in the study are included in the article; further inquiries can be directed to the corresponding author.
